# A new perspective on meals as part of an Optimized Mixed Diet for children and adolescents

**DOI:** 10.3389/fnut.2022.981587

**Published:** 2022-09-06

**Authors:** Mathilde Kersting, Hermann Kalhoff, Kathrin Sinningen, Thomas Lücke

**Affiliations:** ^1^Research Department of Child Nutrition, St. Josef-Hospital, University Hospital of Pediatrics and Adolescent Medicine, Ruhr-University Bochum, Bochum, Germany; ^2^Pediatric Clinic Dortmund, Dortmund, Germany; ^3^St. Josef-Hospital, University Hospital of Pediatrics and Adolescent Medicine, Ruhr-University Bochum, Bochum, Germany

**Keywords:** children, nutrition, Optimized Mixed Diet, nutrients, foods, meals

## Abstract

**Objectives:**

To show by the example of the Optimized Mixed Diet (OMD) for children and adolescents in Germany, how the different food and nutrient profiles of the traditional daily meals complement each other to achieve daily nutrient intakes that meet the Dietary References V.

**Methods:**

The 7-day menu plan of the OMD with the usual 5 daily meals in Germany was used. The total nutrient intake from all meals was compared with the nutrient references. Then the composition of the meals was optimized.

**Results:**

Although the cooked meal (lunch) provides only 25% of the daily energy intake, it is relatively rich (>25% of the daily intake) in most vitamins and minerals, which distinguishes it from the other meals. The cold main meals (breakfast, dinner) are rich in calcium and vitamin B2, due to the preferential use of milk in these meals. The two snacks each provide 12.5% of the daily energy intake.

**Discussion and conclusion:**

People eat foods but not nutrients and they eat foods as meals; this holds especially true for children and adolescents. A well-calculated menu plan can assure the nutrient adequacy of an OMD where the different food and nutrient profiles of the meals complement each other in a modular system. Guidelines for meals could facilitate flexible coordination of family meals and meals in childcare centers and schools. Different meal types set varied stimulus patterns at different levels (neurocognition, emotion, digestion), which may open up long-term health benefits.

## Introduction

The foundations for lifelong health are set in childhood and adolescence. A key requirement is a healthy diet from the beginning, ensuring an adequate energy supply with all the nutrients necessary for health, growth, development, and performance (1, 2). Prevention of diseases later in life, e.g., cardiovascular diseases or type 2 diabetes should be considered as well (3).

Dietary Reference Values (DRV) or Dietary Reference Intakes (DRI), reflect the scientific knowledge about the nutrient needs of the different age groups across the life cycle (4). To become understandable for the population, these nutrient values have to be translated to foods. This is the aim of the so-called Food-Based Dietary Guidelines (FBDG). Besides focusing on foods, FBDG may also take into consideration variables like tradition, dietary culture and the health status of the target groups (5). Usually FBDG inform about food groups, proportions, and general food selection. Typical examples are the 10 rules of the German Nutrition Society (www.dge.de), or the my plate system in the USA (www.choosemyplate.gov). However, in order to realistically calculate the daily nutrient intake for comparison with the nutrient references, the exact amounts of the recommended individual foods in the daily diet must be known.

The German FBDG for children and adolescents, named the Optimized Mixed Diet (OMD) (6), are based on recipes for daily meals, in which the recommended amounts of individual foods are given for certain age ranges (7, 8). The everyday nutrition practice takes place in the daily meals, thus meals provide a true-to-life foundation to quantify the usual daily food intake.

In earlier publications we have shown, that reference values for daily nutrient intake were met by the OMD seven day menu plan in all age groups of children and adolescents (6, 8). Here we show the food and nutrient profiles of the OMD meals and how the different profiles complement each other in a modular system. We discuss the advantages of this approach: 1. to ensure the nutrient adequacy of the total daily diet 2. to enable the practicability of FBDG 3. to better align FBDG with socio-cultural and physiological aspects of meal patterns (e.g., chrono-nutrition).

## Procedures

### Overview

The development of the OMD started with a 7-day menu comprising the usual 5 daily meals for children and adolescents in families in Germany. For an exemplary age group (4–6 years), the average nutrient intake from each of the meals was calculated and the sum of all meals per day was compared with the respective daily reference nutrient values. In this way, potential needs for optimization of food selection or food amounts became visible. Subsequently, meal-specific food and nutrient profiles were obtained (6, 7).

### Foods in meals

#### The 7-day menu

For the 7-day menu plan, both practical and scientific criteria were considered: firstly, adhering to typical meal patterns in families in Germany that comprise 5 daily meals and can be categorized into 3 meal types: 2 cold main meals, 1 cooked meal, 2 snack meals; secondly, using primarily common nutrient-dense, non-fortified foods, to realize the full potential of their health-promoting ingredients while also identifying any need for specific nutrient fortification or supplementation, thirdly, considering the food preferences of children and adolescents. As an overarching guidance, beverages and plant foods should be consumed abundantly, foods of animal origin moderately, and high fat and high-sugar foods sparingly.

#### Examples

- a portion of raw or cooked vegetables or fruits with almost each meal
- cereals preferably as whole grains, breakfast cereals as a mixture of oat flakes and common non-fortified cornflakes
- water as beverage of choice (or unsweetened herbal or fruit tea) at meals and in between meals
- sugary products (so-called “tolerated” foods) limited to morning and afternoon snack at home to facilitate subsequent tooth brushing

The individual foods were grouped in nutritionally and practically useful groups, the amounts per food group and meal of the 7 days were summed and averaged per day (8) (Table 1). From the percentages of the food groups, the appropriate food group amounts for all other age groups can be calculated based on the age-specific total daily energy requirement.

**Table 1:** Proportion of food groups in the daily meals of the OMD.

		**Breakfast**	**Mid-morning snack**	**Lunch**	**Afternoon snack**	**Dinner**	**Total g**
Vegetable/raw food	g (%)	(^a^)	30 (18, 5)	120 (37, 0)	(^a^)	70 (29, 3)	220
Fruit	g (%)	80 (29, 7)	50 (10, 9)	6 (1, 8)	70 (^b^)	10 (4, 1)	216
Bread/cereal flakes	g (%)	45 (16, 7)	35 (21, 6)	10 (3, 1)	-	45 (18, 8)	135
Potatoe/pasta/rice	g (%)	-	-	110 (34, 0)	-	10 (4, 1)	120
Dairy (-products)	g (%)	140 (52, 0)	35 (21, 6)	35 (10, 8)	55 (^b^)	85 (35, 6)	350
Meat/sausage	g (%)	(^c^)	6 (3, 7)	20 (6, 2)	-	5 (2, 1)	31
Eggs	g (%)	2 (0, 7)	3 (1, 9)	7 (2, 2)	-	4 (1, 7)	16
Fish	g (%)	-	-	10 (^d^) (3, 1)	-	-	10
Oil/margarine/butter	g (%)	2 (0, 7)	3 (1, 9)	6 (1, 8)	-	10 (4, 2)	21

^a^Can be exchanged for fruit (same amount). ^b^Variable proportion depending on the selection of “tolerated” foods in this meal. ^c^Can be exchanged for meat/sausage in the snack. ^d^1 time/week (70 g).

#### Food profiles of the meals

In the two main cold meals (breakfast and dinner), milk or dairy products make up the highest proportions of the total meal amount. In addition, fruit or raw vegetables, as well as cereal flakes (as muesli) or a sandwich (bread) are consumed. Practical examples of cold meals are muesli of cereal, fruit and yogurt, a sausage sandwich with a glass of milk and an apple, a cheese sandwich with a raw vegetable salad or a pasta salad with tomatoes, cucumbers and yogurt dressing.

The main components of the cooked meal (typically lunch) are potatoes, rice or noodles, with plenty of vegetables, also as raw vegetable salad. A small portion of meat is on the menu about three times a week, a meal with fish once a week. On the other days, there are vegetarian dishes with pulses or cereals and vegetables as main ingredients.

The two snacks are usually eaten in the morning (e.g., as a second breakfast in the kindergarten or at school) and in the afternoon. They consist mainly of fruit or raw vegetables, bread or cereal flakes and a portion of milk or a dairy product. Occasionally, sweets, cookies or cakes are accepted.

A no-energy drink, preferably tap water, accompanies each meal and should also be available in between.

The energy percentage of the meals in the daily diet was fixed with 25% for each of the main meals (breakfast, lunch, dinner) and 12.5% for each of the two snacks. While absolute food amounts (g/d) vary with age and sex due to varying energy requirements, food group proportions (%) in the meals are independent of age (6) (Table 1).

### Nutrients in meals

#### Calculation of nutrient intake

To calculate the energy and nutrient intake, a common software (DIAT-2020 Soft & Hard, D. Beyer, Rimbach, Germany) was used. Nutrient values were obtained from the German Food Code and Nutrient Data Base (BLS) (Bundeslebensmittelschlüssel, BLS, Version II.3), which has already repeatedly been used for nutrient intake calculations across Europe in the HELENA study (9). The BLS considers nutrient loss due to food preparation, such as cooking vegetables or frying meat.

**Figure 1:**
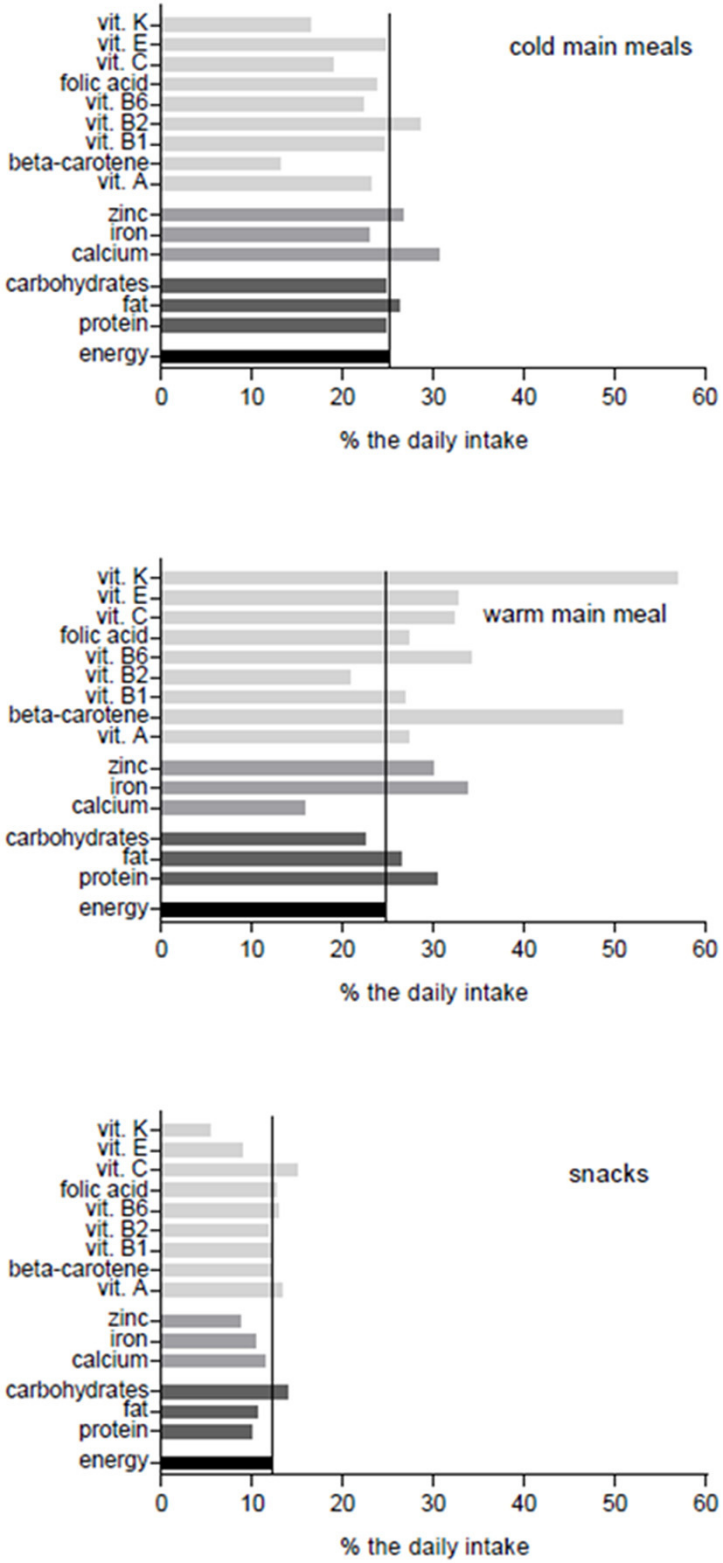
Nutrient profiles of the three meal types depicted as percentage of the daily intake (g, mg/day) as 100%.

Figure 1 shows the contribution of each of the 3 meal types to the daily intake of different nutrients in comparison to the fixed energy proportion of the respective meal types. Although the cooked meal (lunch) provides only 25% of the daily energy intake, it is relatively rich (>25% of the daily intake) in most vitamins and minerals, setting it apart from the other meals.

The cold meals are especially rich in calcium and Vitamin B2, due to the preferential use of milk in these meals. The snacks show a balanced nutrient profile, which is achieved by upgrading the usual afternoon snack of sweets or cakes with the repeated use of cereals, raw vegetables and fruit.

## A new perspective on meals

### Role of meals

People eat foods but not nutrients and they eat foods as meals. Nutrition recommendations are usually given by FBDG in a qualitative or semi-quantitative way for the whole day or even more generally. Meals, with their different profiles, more accurately reflect the daily dietary practices and the various typical eating occasions. Beyond their typical contribution to a total healthy diet, meals provide an important setting for social gathering and for communication (10). The OMD meal is based on optimized common meals and thus could make dietary recommendations more practical and at the same time more precise to ensure adequate nutrient intake while preserving the cultural-historical variability of meals. The role of meals may go beyond the adequacy of nutrient intake, as meals are part of chronobiology of physiological and psychological processes.

### Meals as a source of nutrients

The meals in a mixed diet have specific food profiles that result in specific nutrient profiles. This means that the individual meals cannot, and need not, fully reflect a healthy daily diet on their own. Finally, it is important that the meals form a modular system that completes to a daily nutrient intake that is consistent with the nutrient references.

There is no convincing evidence for a certain number of daily meals for health promotion and disease prevention (11). The OMD meal concept with 3 main meals and 2 snacks is pragmatic, but it is close to the ESPGHAN recommendations of about 4 meals per day for the prevention of childhood obesity (12).

The sequence of meals in the OMD may vary throughout the day depending on the needs of the family or child. For example, the cooked main meal provided as lunch in the OMD menus, can be exchanged for the cold main meal in the evening. The two meals during the morning (breakfast and mid-morning snack) can be interchanged for example in older children who often have a later chronotype than younger children (13) and do not feel much appetite in the morning. The OMD leaves room for such an individualized 'breakfast-combination without compromising nutrient intake (14).

### The role of meals beyond nutrients

Meals are diverse by nature, thus corresponding to cultural food patterns and eating traditions, especially in rich countries. Diversity results from the different shares of food groups in the different daily meals and, in addition, from variability of the foods within a food group on different days, e.g. a cereal breakfast or a bread breakfast on two different days.

Recent work on chronobiology, pleasure of eating and long-term physical health suggests benefits of diversity between and within meals. For example, since physiological mechanisms are generally subject to diurnal regulation, it is to be expected that the time of day is also important for postprandial physiology. This may have consequences not only for wellbeing but for risk reduction and long-term health (15, 16).

Additionally, differentiated meal compositions help to anticipate and remember mealtime as a social experience and a joyful event (17, 18).

Children grow into these specific roles and meanings of meals in the family. They could probably benefit from a broader view of meals. For example, hedonistic attitudes toward food have been hypothesized to promote healthier food choices in children compared with food-related attitudes (19).

### Implementation of healthy meals for children and adolescents

The breakdown of FBDG into meals to facilitate practical nutritional counseling may cover different social areas. For example, communicating healthy eating through meals could be of practical importance for children and adolescents. who eat meals outside the family in day care centers (younger children) and schools (older children, adolescents) (20).

The OMD's modular meal system shows that it is not acceptable in a mixed diet to set standardized nutrient proportions for individual meals, e.g., 1/3 of the daily nutrient references for each hot meal in day care or school, without jeopardizing the balanced daily nutrient intake of the modular system.

It is the diversity of meals that facilitates balance in daily nutrient intake. The OMD makes this possible while maintaining the meal habits in Germany. In the case of changes in meal habits (e.g., by internationalization of guidelines), adjustments could easily be made and nutrient adequacy evaluated.

## Conclusion

People eat foods, but not nutrients, and they eat foods as meals. Using the example of the meals of the OMD in Germany, the results provide benchmarks for the composition of all meals of the day for children and adolescents while preserving the existing nutrition culture. Meal-Based Dietary Guidelines could facilitate the flexible coordination of family meals with daycare and school food service to achieve a balanced daily diet in a practical way. Different meal types also open up the possibility of setting varied stimulus patterns at different levels (neurocognition, emotion, digestion), which not only lead to more enjoyment when eating, but can also open up long-term health benefits.

## Data availability statement

The raw data supporting the conclusions of this article will be made available by the authors, without undue reservation.

## Author contributions

MK designed the study. MK, HK, and KS analyzed data and drafted the manuscript. TL supervised the project. All authors contributed to interpretation of the data and revisions of the manuscript.

## Conflict of interest

The authors declare that the research was conducted in the absence of any commercial or financial relationships that could be construed as a potential conflict of interest.

## Publisher's note

All claims expressed in this article are solely those of the authors and do not necessarily represent those of their affiliated organizations, or those of the publisher, the editors and the reviewers. Any product that may be evaluated in this article, or claim that may be made by its manufacturer, is not guaranteed or endorsed by the publisher.

## References

[1] Robinson S Fall C. Infant nutrition and later health: a review of current evidence. Nutrients. (2012) 4:859–74. 23016121 10.3390/nu4080859 PMC3448076

[2] Das JK Lassi ZS Hoodbhoy Z Salam RA. Nutrition for the next generation: older children and adolescents. Ann Nutr Metab. (2018) 72:56-64. 29631269 10.1159/000487385

[3] Heindel JJ Vandenberg LN. Developmental origins of health and disease: a paradigm for understanding disease etiology and prevention. Curr Opin Pediatr. (2015) 27:248–53. 25635586 10.1097/MOP.0000000000000191 PMC4535724

[4] European Food Safety Authority Dietary Reference Values for nutrients. Summary Report. EFSA Supporting Publication. p. 98.

[5] Herforth A Arimond M Álvarez-Sánchez C Coates J Christianson K Muehlhoff E. A global review of food-based dietary guidelines. Adv Nutr. (2019) 10 590–605. 31041447 10.1093/advances/nmy130 PMC6628851

[6] Kersting M Alexy U Clausen K. Using the concept of food based dietary guidelines to develop an optimized mixed diet (OMD) for German children and adolescents. J Pediatr Gastroenterol Nutr. (2005) 40:301–8. 15735483 10.1097/01.mpg.0000153887.19429.70

[7] Kersting M Chahda C Schöch G. Optimised mixed foods as a feeding for children and adolescents. Part 1. Food selection, part 2. Nutrient intake, Part 3. Meal plans. Nutr Rev. (1993) 40:164–169.

[8] Kersting M Kalhoff H. Lücke T. From nutrients to food and meals: the concept of the optimized mixed diet for children and adolescents in Germany (Article in German). Aktuel Ernahrungsmed. (2017) 42:304–15.

[9] Vanhelst J Béghin L Duhamel A. Physical activity awareness of European adolescents: the HELENA study. J Sports Sci. (2018) 36:558–64. 28481665 10.1080/02640414.2017.1323116

[10] Dunbar RIM. Breaking Bread: the Functions of Social Eating. Adapt Human Behav Physiol. (2017) 198–211. 32025474 10.1007/s40750-017-0061-4 PMC6979515

[11] Kaisari P Yannakoulia M Panagiotakos DB. Eating frequency and overweight and obesity in children and adolescents: a metaanalysis. Pediatr. (2013) 131:958–67. 10.1542/peds.2012-3241 23569087

[12] Agostoni C Braegger C Decsi T. Role of dietary factors and food habits in the development of childhood obesity: a commentary by the ESPGHAN Committee on Nutrition. J Pediatr Gastroenterol Nutr. (2011) 52:662–9. 21593641 10.1097/MPG.0b013e3182169253

[13] Roenneberg T Kuehnle T Pramstaller PP. A marker for the end of adolescence. Curr Biol. (2004) 14:1038–9. 10.1016/j.cub.2004.11.039 15620633

[14] Research Institute for Child Nutrition. Recommendations for Breakfast: The Breakfast Twice With optiMIX, Dortmund: FKE Own Publication. (2014).

[15] Johnston JD. Physiological responses to food intake throughout the day. Nutr Res Rev. (2014) 27:107–8. 24666537 10.1017/S0954422414000055 PMC4078443

[16] Katsi V Papakonstantinou IP Soulaidopoulos S Katsiki N Tsioufis K. Chrononutrition in cardiometabolic health. J Clin Med. (2022) 7:296. 10.3390/jcm11020296 PMC8780356 35053991

[17] Kringelbach M.L. The pleasure of food: underlying brain mechanisms of eating and other pleasures. Flavour. (2015) 4:20.22487544

[18] Cipriano-Crespo C Rivero-Jiménez B Conde-Caballero D Medina FX Mariano-Juárez L. The denied pleasure of eating: a qualitative study with functionally diverse people in Spain. Foods. (2021)10:628. 33809545 10.3390/foods10030628 PMC7998917

[19] Marty L Miguet M Bournez M Nicklaus S Chambaron S Monnery-Patris S. Do hedonic- versus nutrition-based attitudes toward food predict food choices? A cross-sectional study of 6- to 11-year-olds. Int J Behav Nutr Phys Act. (2017) 14:162. 29178916 10.1186/s12966-017-0618-4 PMC5702150

[20] Briley M Mc Allaster M. Nutrition and the child-care setting. J Am Diet Assoc. (2011) 111:1298–300. 21872691 10.1016/j.jada.2011.06.012

